# Who uses legal cannabis and why? Cluster profiles of participants in a Swiss regulated sales pilot trial

**DOI:** 10.1016/j.dadr.2026.100445

**Published:** 2026-05-19

**Authors:** Maëlle Dreifuss Bisson, Tamara Corino, Tatiana Aboulafia Brakha, Federico Seragnoli, Laura Toso, Sandro Cattacin, Daniele Zullino, Lucas Spierer, Lucien Rochat

**Affiliations:** a Division of Addictology, Psychiatry Department, Geneva University Hospitals, Geneva, Switzerland; b Department of Sociology, University of Geneva, Geneva, Switzerland; c Faculty of Medecine, University of Fribourg, Fribourg, Switzerland; d Faculty of Psychology, UniDistance, Switzerland

**Keywords:** Cannabis regulation, Motives, Risk perception, Cluster analysis, Harm reduction, Cannabis use disorder

## Abstract

**Background:**

As cannabis regulation expands, it is important to understand who enrolls in legal non-medical cannabis programs and how participant profiles relate to harm. We identified participant profiles in Geneva’s pilot trial, *La Cannabinothèque*, using motives for cannabis use, knowledge of lower-risk use, and perceived risk, and examined differences in key outcomes.

**Methods:**

Baseline data from 1290 people who use cannabis (PWUC) were analysed. Hierarchical clustering (Ward) followed by K-means refinement supported a four-cluster solution (Cramer’s V = 0.563). Clusters were compared on problematic cannabis use and consumption frequency, mental health symptoms, and quality of life.

**Results:**

Cluster 1 (*High motives – Low safer-use knowledge – Moderate risk perception*; 9.9%) showed high endorsement of all motives, lower-risk knowledge, and moderate to elevated perceived risk, and had the highest problematic use/frequency and poorer health indicators. Cluster 2 (*Moderate motives – Average safer-use knowledge – Low risk perception*; 26.7%) had moderate motives (relatively higher enhancement/social), average knowledge, lower perceived risk, and the lowest problematic use with better health indicators. Cluster 3 (*Low motives – High safer-use knowledge – High risk perception*; 29.4%) combined low-to-average motives with the highest knowledge and perceived risk, yet showed frequent use and problematic use comparable to Cluster 1. Cluster 4 (*Minimal motives – Low safer-use knowledge – Very low risk perception*; 34.0%) reported minimal motives, low knowledge and perceived risk, and low use/cannabis-related harms; members were older with later initiation.

**Conclusions:**

Findings support the link between coping-related motivation and problematic use, but the high-risk profile of Cluster 3 despite strong knowledge and risk awareness suggests that information and perception alone may not reduce harm. Profile-tailored harm reduction and integrated support may be needed within regulated cannabis frameworks.

## Highlights

• Four cannabis use profiles identified in a Swiss regulated sales pilot.
• Profiles differed in use motives, perceived risk, and harm-reduction knowledge.
• Coping-related use was associated with higher problematic cannabis use.
• High perceived risk did not consistently align with lower use levels.
• Findings inform targeted prevention in regulated cannabis markets.

## 1 Introduction

Cannabis is the most widely used illegal drug globally, with an estimated 219 million people aged 15–64 reporting use in 2021 (4.3% of the adult population; UNODC, 2023). Switzerland broadly mirrors these global patterns: in 2022, 7.6% of those aged 15–64 reported past-year use and 4.0% past-month use, with higher prevalence among men and young adults (FOPH/BAG, 2023; Federal Statistical Office [FSO/OFS], 2022; Lociciro et al., 2019). While some countries have legalized recreational use, Switzerland authorized medical cannabis only in 2022 (Snapp et al., 2023). At the same time, prohibition has proven ineffective in curbing use or dismantling the black market (Herzig et al., 2019). In this context, the Federal Office of Public Health, 2023 (FOPH) has launched tightly controlled pilot trials to evaluate the impact of regulated sales of non-medical cannabis (FOPH/BAG, 2023). Beyond prevalence, cannabis use carries well-documented risks. A substantial minority of people who use cannabis develop cannabis use disorder (Leung et al., 2020). Heavier and more frequent use has also been associated with poorer mental health and quality of life, psychotic symptoms in vulnerable individuals, and worse educational and occupational outcomes (Compton et al., 2004, Lev-Ran et al., 2012, Liao et al., 2019, Taylor et al., 2019). Cannabis use is also associated with impaired driving and broader social harms (Fischer et al., 2017, Hall and Weier, 2015).

Understanding who engages in such regulated access models is therefore important from a public health perspective. Because these harms concentrate in specific subgroups rather than being evenly distributed across PWUC (Leung et al., 2020, Taylor et al., 2019), regulated frameworks need not only to document participation but also to characterize whether different participant profiles map onto different levels of harm.

Several psychological and behavioral factors may help explain why some cannabis PWUC experience greater harms than others within regulated access frameworks. Among the most relevant are motives for use, knowledge of lower-risk practices, and perceived risk. Of these, motives for cannabis use play a central role in shaping consumption behaviors and related risks. The Five-Factor Marijuana Motives Model (Simons et al., 1998) is widely used to assess these motives (coping, enhancement, social, expansion, conformity). Coping, using cannabis to reduce negative affect or manage stress, shows the strongest and most consistent links to problematic use: it is associated with more frequent use and poorer mental health, and is prospectively associated with later cannabis-related harms (Glodosky and Cuttler, 2020, Moitra et al., 2015, Moitra et al., 2021, Rochat et al., 2024). Mechanistically, coping aligns with negative-reinforcement/self-medication accounts whereby transient relief reinforces use; over time, tolerance and withdrawal symptoms become additional triggers, and avoidant emotion regulation fosters solitary, heavier patterns (Baker et al., 2004, Khantzian, 1997). Daily-level data converge with this view as on days when coping is higher, quantities consumed increase (Bonar et al., 2017).

Beyond coping, enhancement and social motives are robustly associated with higher use frequency and quantity; expansion likewise tracks frequency, whereas conformity is often weakly or negatively related to frequency when adjusted for other motives yet positively associated with harms when endorsed (Bresin and Mekawi, 2019, Bonar et al., 2017). Because these motives co-occur and confer distinct risk profiles, a person-centered approach (clusters/profiles) can capture heterogeneous motive combinations that map onto different risk levels and intervention targets.

In addition to motives, knowledge of lower-risk cannabis use and perceived risk are also relevant to understanding cannabis-related outcomes. As cannabis use increases, PWUC knowledge of lower-risk practices becomes critical for promoting safer consumption. In the context of the present study, knowledge of lower-risk cannabis use refers to safer-use practices in line with national and international Lower-Risk Cannabis Use Guidelines (Fischer et al., 2017), covering key domains including preferred routes of administration (e.g., avoiding smoking), THC/CBD content awareness, avoiding mixed use with alcohol or tobacco, refraining from driving under the influence, and delaying onset of use. Such knowledge enables people who use cannabis (PWUC) to make informed decisions that balance benefits with risks (Fischer et al., 2017, Volkow et al., 2014). Perceived risk refers to participants’ subjective appraisal of the health risks associated with their current cannabis use pattern. Declining perceived risk has been associated with increased use and a higher likelihood of cannabis use disorder (Ramirez et al., 2020, Salloum et al., 2018). Evidence points to bidirectional associations: higher cannabis use has been associated with subsequent decreases in perceived risk over time, whereas higher risk perception is associated with lower use. Frequent PWUC often exhibit optimism bias, underestimating their risks (Weinstein et al., 2005).

The present study draws on baseline data from *La Cannabinothèque*, a pilot trial of regulated non-medical cannabis sales conducted in Geneva. The primary objective was to identify distinct participant profiles based on motives for cannabis use, knowledge of lower-risk use, and perceived risk. A secondary objective was to examine whether these profiles differed on external correlates, including problematic cannabis use, consumption frequency, quality of life, psychiatric symptoms, and sociodemographic characteristics.

Although exploratory, the study was guided by two hypotheses. First, based on prior evidence linking coping motives to problematic use and poorer mental health, we expected clusters characterized by elevated coping motives to show greater problematic use, lower quality of life, and more psychiatric symptoms (e.g., Bresin and Mekawi, 2019; Glodosky and Cuttler, 2020). Second, because greater knowledge of lower-risk practices and higher perceived risk are generally associated with fewer cannabis-related harms, we expected clusters scoring high on these dimensions to report less frequent and less problematic cannabis use (Fischer et al., 2017, Volkow et al., 2014).

## 2 Materials and methods

### 2.1 Recruitment

*La Cannabinothèque*, launched in December 2023, is a three-year pilot project funded by the non-profit ChanGE association, which oversees both local cannabis production and its regulated distribution. The project’s primary aim is to provide participants with access to high-quality, organically grown cannabis with controlled THC levels, ranging from 2% to 20% THC. Purchases are limited to a maximum of 10 g per visit and up to 10 g of pure THC per month. Data refer to participants enrolled between October 9, 2023, and February 12, 2025.

Interested individuals registered online to schedule an inclusion interview with a clinical psychologist. Under the Swiss Ordinance on Pilot Trials under the Narcotics Act (OEPStup), eligibility was assessed based on the following inclusion criteria: age ≥ 18 years, ability to provide informed consent, sufficient knowledge of French, residency in the Canton of Geneva, and recreational cannabis use at least once per month in the preceding six months. Exclusion criteria included current pregnancy or breastfeeding, severe medical or psychiatric conditions and having received addiction treatment for alcohol or other substances (excluding tobacco) in the past 12 months (OEPStup, art. 14; FOPH FAQ). These eligibility rules reflect FOPH youth- and health-protection objectives by limiting enrollment to adult existing users and excluding situations with increased health risk.

### 2.2 Participants

A total of 1365 individuals attended the inclusion interview; 1290 were included. Exclusions were mainly due to ineligibility (notably for psychiatric conditions or recent addiction treatment) or non-completion of baseline questionnaires.

The mean age of participants was 38.1 years (SD = 12.9; range: 18–79 years). Of the sample, 21.4% identified as female, 76.8% as male, and 1.8% as non-binary, transgender (female or male), or other gender identities.

Living arrangements were: alone (30.5%), couple without children (20.5%), family households (41.2%), and other shared arrangements (7.8%). Education: no formal or only compulsory education (7.8%), vocational training or a high school diploma (35.0%), and higher education (57.2%). Employment: employed full- or part-time (65.8%), unemployed (9.8%), in training (12.1%), and economically inactive (12.2%). Monthly household income (in Swiss francs, CHF): less than CHF 3000 (37.0%), between CHF 3000 and 6500 (38.1%), and more than CHF 6500 (24.9%).

### 2.3 Procedure and ethics

This is an observational longitudinal study with assessments conducted every six months over three years. After confirming eligibility, participants received verbal and written information, provided written informed consent, and completed the baseline assessment (T0) via online self-report questionnaires distributed through the REDCap® platform. This manuscript focuses on data collected at baseline; follow-up data will be analyzed and reported in subsequent publications. The study complies with the Declaration of Helsinki and was approved by the Ethics Committee of the Canton of Geneva (BASEC-ID 2017–01191).

### 2.4 Measures

#### 2.4.1 Cannabis use disorder identification test-revised (CUDIT-R)

The CUDIT-R (Adamson et al., 2010; French version: Luquiens et al., 2021) is an 8-item screening tool used to assess problematic cannabis use (PCU) over the past six months. Items are rated on a Likert scale from 0 to 4 (total score: 0–32). A score of ≥ 8 indicates hazardous use, and a score of ≥ 12 suggests a potential cannabis use disorder (CUD) warranting further clinical evaluation. The CUDIT-R is widely validated and used in both clinical and research settings (Schultz et al., 2019). Given concerns that item 1 (frequency of use) may disproportionately drive the total CUDIT-R score, we additionally computed a modified score excluding item 1 (CUDIT-1) and examined its correlations with cannabis use frequency, item 1, and the original CUDIT-R total to assess potential redundancy.

#### 2.4.2 Patterns of Cannabis consumption

Patterns of cannabis use were assessed via self-report frequency of use (5-point Likert-type scale from “never” to “4 times per week or more”) and age at first use (single open-ended item).

#### 2.4.3 Motivations for participation

This 7-item questionnaire was developed by the FOPH (2023) specifically for cannabis pilot trials. It assesses participants’ motivations for joining the study (e.g., easier access to cannabis, better product quality, greater variety, interest in the research, curiosity, and a desire to contribute to new regulatory models). Items are rated on a 5-point Likert scale from “Absolutely false” to “Absolutely true.”

#### 2.4.4 Marijuana Motives Measure (MMM)

The MMM (Simons et al., 1998; French version: Chabrol et al., 2005) is a 25-item self-report questionnaire measuring motives for cannabis use. It captures five dimensions: Coping, Social, Enhancement, Conformity, and Expansion. Coping describes the use of cannabis to manage or alleviate unpleasant emotional states (e.g., “to forget my problems or my worries”). Enhancement refers to consuming the substance to intensify pleasurable feelings or experiences (e.g., “because it’s fun,”). Social motive refers to using cannabis in the company of others to amplify enjoyment in social contexts (eg, “because it improves parties or social gatherings”). Conformity captures cannabis use aimed at maintaining group belonging and avoiding exclusion (eg, “to fit in with the group”). Expansion reflects using cannabis to broaden one’s consciousness or perspective (eg, “to expand my awareness,” “to understand things differently”). Items are rated on a 5- point Likert scale (1 = almost never/never to 5 = almost always/always) and summed to obtain subscale scores. Higher scores reflect stronger endorsement of specific motives. The French version has demonstrated adequate psychometric properties in francophone populations (Chabrol et al., 2005)

#### 2.4.5 Lower-Risk Cannabis Use – Knowledge Scale (LRCU-K)

Knowledge of safer cannabis use was assessed with the Lower-Risk Cannabis Use – Knowledge Scale (LRCU-K), a 12-item scale developed for the Swiss regulated cannabis pilot trials to operationalize “consumption competence” in line with national safer-use recommendations and international Lower-Risk Cannabis Use Guidelines (Gubser and Nordt, 2022). Items cover key safer-use recommendations (e.g., THC/CBD-related risks, mixed use, driving, tobacco-related risks). Responses are rated on a 5-point scale and recoded from 0 to 4; item scores are summed (range 0–48), with higher scores indicating greater knowledge of lower-risk cannabis use. Initial validation suggests moderate internal consistency (Cronbach’s α ≈ 0.55–0.62) with further validation ongoing in the context of Swiss cannabis pilot trials.

#### 2.4.6 Perceived risk associated with cannabis use

Perceived health risk of cannabis use was measured with one item (Gubser and Nordt, 2022): “How do you rate the health risks of your current cannabis use?”, rated on an analog scale from 0 to 100. Higher scores indicate greater perceived health risks related to one’s current cannabis use. Although this single-item measure does not specify the type of risk considered, global risk perception has been consistently shown to predict cannabis use behaviors and related outcomes (Salloum et al., 2018, Ramirez et al., 2020), supporting its inclusion as a clustering variable despite its broad framing.

#### 2.4.7 EuroQol Five-Dimension Visual Analogue Scale (EQ-5D VAS)

For this study, only the EQ-5D visual analogue scale (VAS) component was used. Participants rate their current health status from 0 (worst imaginable health) to 100 (best imaginable health).

#### 2.4.8 Modified Mini Screen (MMS)

The MMS (Alexander et al., 2008) is a 22-item screening tool for identifying possible mood, anxiety, and psychotic disorders. Items are answered with “yes” or “no," and a total score of 6 or more suggests the likely presence of a psychiatric disorder warranting further evaluation.

### 2.5 Statistics

To identify subgroups of participants based on motives to use cannabis, risk perception, and knowledge of low-risk cannabis use, cluster analyses followed Clatworthy et al. (2005). The analysis began with a hierarchical agglomerative method (Ward’s method) using squared Euclidean distance; the number of clusters was selected by examining the dendrogram and agglomeration coefficients. K-means clustering then refined the solution. Cluster stability was assessed by comparing Ward and K-means solutions using Cramer’s V. To further validate the four-cluster solution, multiple internal validation indices were computed for solutions ranging from k = 2 to k = 6, including the Calinski-Harabasz index and the Davies-Bouldin index. Cluster stability was additionally assessed using bootstrap validation (B = 10,000 resamples) with Jaccard coefficients (Hennig, 2007), implemented via the clusterboot function in R. Cluster comparisons on external correlates (PCU, pattern of use, comorbidities, quality of life, sociodemographic) used ANOVA for continuous data, Kruskal-Wallis tests for ordinal data or when assumptions were not met, and χ² tests for categorical variables, with Games-Howell or Mann-Whitney U post hoc tests as appropriate. To control for Type I errors, a significance level of p < .001 was applied. All statistical analyses were two-tailed.

## 3 Results

### 3.1 Preliminary analyses

A pronounced floor effect was observed for the Conformity motive on the Marijuana Motives Measure (MMM), as participants reported consistently low endorsement of this factor across the sample. Similarly, a ceiling effect was noted for the study participation motives scale: most participants expressed high agreement with items related to product quality, variety of choice, ease of access, interest in the study and its results, and the desire to contribute to future regulatory models (see Appendix A). Consequently, the Conformity factor and the participation motives scale were excluded from further cluster analyses.

Internal consistency estimates ranged from acceptable reliability for the Lower-Risk Cannabis Use – Knowledge scale (LRCU-K; McDonald’s ω =.68) to excellent reliability for the expansion factor of the MMM (ω =.90).

Spearman correlations among the six clustering variables are presented in Appendix C. The four cannabis use motives showed low to moderate positive intercorrelations (ρ =.17–.47), while knowledge of lower-risk use and perceived risk were largely independent of the motives (ρ = −.09–.16) and weakly correlated with each other (ρ =.21). These patterns support the use of all six variables as distinct contributors to the cluster solution and are consistent with prior research showing that motives, harm reduction knowledge, and risk perception represent separate psychological constructs in cannabis PWUC (Lee et al., 2007, Pedersen et al., 2016).

## 4 Cluster analysis

All variables entered the cluster analysis were z-transformed to ensure a common metric and equal contribution to cluster formation.

A hierarchical cluster analysis was first conducted using Ward’s method with squared Euclidean distance as the dissimilarity measure. Visual inspection of the dendrogram and the agglomeration schedule suggested an optimal four-cluster solution. To refine and confirm this solution, a non-hierarchical K-means cluster analysis was performed, constrained to four clusters. The results showed good agreement between the hierarchical and K-means methods, as evidenced by a Cramer’s V of 0.563 (*p* < .001), indicating a strong association between cluster membership across methods.

Internal validation indices computed for k = 2 to k = 6 were consistent with this choice. The Calinski-Harabasz index showed acceptable separation for k = 4 (267.11) relative to adjacent solutions (k = 3: 305.27; k = 5: 251.35), and the Davies-Bouldin index was moderate across all solutions (range: 1.699–1.837), with k = 4 (1.759) performing better than k = 3 (1.837).

Bootstrap validation (B = 10,000 resamples) yielded Jaccard coefficients ranging from 0.609 to 0.844. Cluster 3 reached the conventional threshold for a stable, reproducible cluster (Jaccard = 0.844; dissolved in only 61 out of 10,000 bootstrap samples; Hennig, 2007). Clusters 1, 2, and 4 fell in the 0.60–0.75 range, which Hennig (2007) interprets as indicating a detectable pattern in the data rather than fully stable clusters. This moderate stability is consistent with the overlap of psychological constructs underlying the clustering variables and with the exploratory nature of the analysis.

**Fig. 1:**
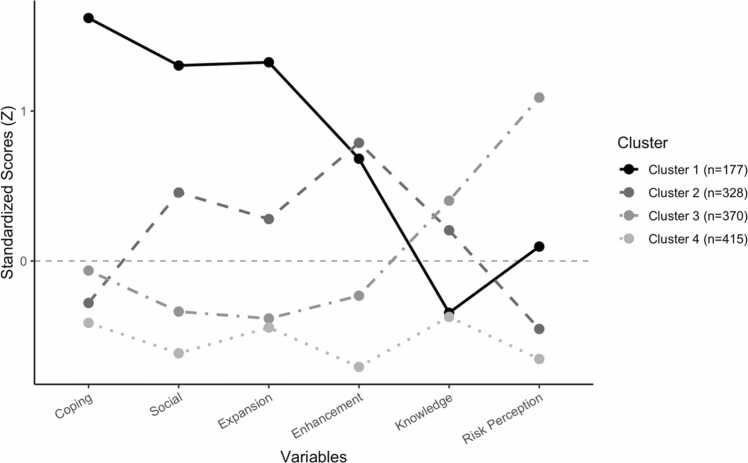
Cluster Profiles Based on Z-Transformed Variables. The four-cluster solution was derived using hierarchical clustering (Ward’s method) followed by K-means refinement. Agreement between methods was strong (Cramer’s V = 0.563, p < .001), and ANOVAs on the profiling variables indicated moderate to large between-cluster effects (partial η² =.09–.51; see Appendix B), supporting the adequacy of the four-cluster solution.

The final four-cluster solution revealed distinct participant profiles, as illustrated in Fig. 1.

ANOVA revealed a significant effect of cluster membership on all factors included in the clustering procedure. Across profiling variables, between-cluster effect sizes were in the moderate to large range (η² =.09–.51; see Appendix B), supporting the distinctiveness of the four profiles**.** Descriptive statistics for each cluster, including means, standard deviations F-values, significance levels, effect sizes, and post hoc comparisons are presented in Appendix B.

Cluster 1, labeled *High motives – Low safer-use knowledge – Moderate risk perception* (N = 128; 9.92% of the total sample), was characterized by high scores across all four Marijuana Motives Measure (MMM) factors—coping, social, expansion, and enhancement. This cluster also displayed lower knowledge of lower-risk cannabis use and a somewhat elevated perception of personal risk.

Cluster 2, labeled *Moderate motives – Average safer-use knowledge – Low risk perception* (N = 345; 26.74%), showed moderate endorsement across the four MMM motives, with relatively stronger scores on enhancement and social motives.

Knowledge of lower-risk use was close to the sample average, while perceived risk was slightly reduced.

Cluster 3, labeled *Low motives – High safer-use knowledge – High risk perception (*N = 379; 29.38%), exhibited low to average levels across all MMM motives, higher knowledge of lower-risk cannabis use, and the highest perceived risk among all clusters.

Cluster 4, labeled *Minimal motives – Low safer-use knowledge – Very low risk perception (*N = 438; 33.95%), reported consistently low levels on all MMM motives. This group also had lower knowledge of lower-risk cannabis use and lower perceived risk.

## 5 Group comparisons on external correlates

Descriptive statistics and group comparisons on external correlates by cluster are presented in Table 1.

**Table 1:** Descriptive statistics, Kruskal-Wallis (H) tests, and χ²analyses on external correlates.

		**Cluster 1**	**Cluster 2**		**Cluster 3**		**Cluster 4**							
		**(N=128)**	**(N=**		**(N=**		**(N=438)**							
			**345)**		**379)**									
**Variable**		**M (SD)**	**M (SD)**		**M (SD)**		**M (SD)**			**H(3)**	**p**		η²KW	
CUDIT-R		11.84	8.64		11.11		7.79			H = 168.83	<		0.13	
		(5.29)^b,d^	(3.68)^a,c,d^		(4.26)^b,d^		(3.42)^a,b,c^				.001		(moderate)	
Frequency of use		4.0 [4.0-	3.0 [2.0-		4.0 [4.0-		3.0 [2.0-			H = 122.43	<		0.09	
		4.0]^b,d^	4.0]^a,c^		4.0]^b,d^		4.0]^a,c^				.001		(moderate)	
Age at first cannabis		17.80	16.97		16.48		17.88			H = 24.99	<		0.02	
use		(6.92)	(4.00)^d^		(3.07)^d^		(5.02)^b,c^				.001		(small)	
MMS		2.06 (2.68)^b,c,d^	0.81 (1.43)^a^		1.10 (1.84)^a^		0.88 (1.72)^a^			H = 37.96	< .001		0.03 (small)	
EQ-5D VAS		80.65	86.41		81.46		85.09			H = 34.38	<		0.02	
		(14.38)^b,d^	(11.12)^a,c^		(14.87)^b,d^		(13.25)^a,c^				.001		(small)	
Perceived risk^†^		35.20 (24.10)	22.20 (15.60)		58.70 (14.80)		17.50 (13.40)			-	-		-	
Age		36.84	35.59		37.56		40.91			H = 35.77	<		0.03	
		(13.53)^d^	(12.23)^d^		(12.08)^d^		(13.45)^a,b,c^				.001		(small)	
Men/Women (%)		78/22	80/20		79/21		72/28			χ² = 3.96	.27		-	
Education		2.0 [2.0-	3.0 [2.0-		3.0 [2.0-		3.0 [2.0–3.0]			H = 8.35	0.039		0.0 (small)	
		3.0]^b^	3.0]^a^		3.0]									
Income		1.0 [1.0-	2.0 [1.0-		2.0 [1.0-		2.0 [1.0-			H = 20.63	<		0.01	
		2.0]^c,d^	2.0]^d^		3.0]^a^		3.0]^a,b^				.001		(small)	
*Type of*										χ² = 21.20	.05			
*household (%)*														
Single-person		33.6	27.0		28.2		34.2			---				
household														
Couple without		14.8	19.1		21.6		21.9			---				
children														
Family household		43.8	42.6		42.2		38.6			---				
Other multi-person households	7.8			11.3		7.9		5.3	-		-			-
*Professional activity (%)*									χ² = 18.42			.03		
Employed full- or	59.4			64.9		66.5		67.8	-		-			-
part-time														
									-		-			-
Unemployed	14.1			9.9		11.3		7.3						
In education or	11.7			15.7		11.6		10.0	-		-			-
training														
Economically	14.8			9.6		10.6		14.8	-		-			-

Inactive

*Note*. EQ-5D VAS: EuroQol 5-Dimension Visual Analogue Scale; MMS: Modified Mini Screen; CUDIT-R: Cannabis Use Disorder Identification Test-Revised. Numbers in the same row that do not share the same superscripts differ at p < .001. ^a^ Significantly different from Cluster 1. ^b^ Significantly different from Cluster 2. ^c^ Significantly different from Cluster 3. ^d^ Significantly different from Cluster 4. Effect sizes (η²KW) were estimated using the Kruskal–Wallis-based eta squared formula and are interpreted as small (.01), moderate (.06), or large (.14). Values are reported as mean and standard deviation, except for the ordinal variables. For these, median and interquartile range [IQR] are reported, while % are reported for categorical variables.

^†^Perceived risk was one of the clustering variables (overall: M = 32.97, SD = 23.63, range: 0–100); between-cluster differences are therefore expected by design and no inferential test is reported. Group comparisons were performed using Kruskal–Wallis tests for continuous and ordinal variables, and chi-squared tests for categorical variables.

Problematic cannabis use and frequency of consumption were significantly higher in Cluster 1 and Cluster 3 compared to Cluster 2 and Cluster 4, with moderate effect sizes for both outcomes (CUDIT-R: η²KW = 0.13; frequency of use: η²KW = 0.09). Participants in Clusters 2 and 4 reported better self-rated health and lower psychiatric symptom scores, with small effect sizes (EQ-5D VAS: η²KW = 0.02; MMS: η²KW = 0.03). These differences were consistent with the cluster profiles, as Clusters 2 and 4 were characterized by lower cannabis use motives and lower problematic use patterns.

Age at first cannabis use was significantly higher in Cluster 4 than in Cluster 3 (small effect; η²KW = 0.02). In terms of demographic characteristics, Cluster 4 participants were older than those in Clusters 2 and 3 and reported higher income than participants in Cluster 1, both associated with small effect sizes (age: η²KW = 0.03; income: η²KW = 0.01).

No significant differences were observed between clusters in gender, household type, educational level, or employment status.

## 6 Robustness checks for the CUDIT-R

Given concerns that item 1 of the CUDIT-R (frequency of use) may disproportionately drive the total score, we conducted robustness checks to assess potential redundancy with the frequency outcome. Spearman correlations showed that cannabis use frequency and CUDIT-R item 1 were strongly associated (ρ =.91), whereas item 1 and the total CUDIT-R score were only moderately correlated (ρ =.49), and frequency was similarly moderately associated with the total score (ρ =.51). By contrast, the total CUDIT-R and a modified score excluding item 1 (CUDIT–1) were almost perfectly correlated (ρ =.97), indicating that item 1 does not disproportionately account for variance in the total score. When we repeated the cluster comparisons using the CUDIT–1 score in Kruskal–Wallis tests, the overall effect remained significant (H = 138.41, df = 3, p < .001), and the rank-ordering of clusters was unchanged (Cluster 1 > Cluster 3 > Cluster 2 > Cluster 4). These findings suggest that our conclusions regarding subgroup differences in cannabis-related problems are robust to the exclusion of the frequency item.

## 7 Discussion

Based on baseline data from 1290 participants enrolled in Geneva’s cannabis pilot program, the present study explored distinct psychological profiles among adult recreational cannabis PWUC enrolled in a regulated sales pilot trial and examined whether these profiles differed on key indicators of harm.

Cluster analyses identified four profiles combining motives for cannabis use, knowledge of lower-risk use, and perceived risk. Two profiles (Clusters 1 and 3) concentrated higher risk, albeit for different reasons, highlighting the value of profile-sensitive prevention and care within regulated markets.

Supporting our first hypothesis, Cluster 1 (high coping motives) showed the highest CUDIT-R scores (with Cluster 3), more frequent use, poorer quality of life, and higher psychiatric symptom levels. High endorsement across other motives suggests a multifunctional pattern of use (West et al., 2024), consistent with emotion regulation PWUC and self-medication. Despite elevated perceived risk, their safer-use knowledge was only average-to-lower, suggesting that general awareness of harm may not translate into detailed harm-reduction practices. This may reflect reduced access to, or lower uptake of, practical safer-use information, and possibly ambivalence about continuing use despite perceived harms (Alvarez-Roldan et al., 2023). Lower income further points to socioeconomic vulnerability, where cannabis may serve as an accessible coping tool. Regulated sales may therefore formalize an existing coping strategy while offering safer, more predictable products. This profile supports integrating harm reduction with mental health-oriented support (for example, motivational interviewing, coping-skills training, and facilitated pathways to care when psychiatric symptoms are elevated).

Cluster 2 reported moderate motives (relatively stronger enhancement and social motives), the lowest problematic use, and the highest quality of life, consistent with a more recreational, socially integrated profile (Ghvanidze et al., 2024). This differs from Cluster 1, where social and enhancement motives co-occur with very elevated coping motives and impairment. Cluster 2 showed average safer-use knowledge and reduced perceived risk, echoing work showing that even informed PWUC may downplay risks of frequent or high-potency use (McMahon et al., 2023). For this group, prevention could leverage peer norms and provide low-friction feedback on frequency, potency, and routes of administration, including reinforcing protective behavioral strategies. Monitoring changes over time remains important, as gradual increases in exposure can translate into harms even among initially low-risk consumers.

Cluster 3 combined high safer-use knowledge and the highest perceived risk with low to average motives, yet showed frequent use and CUDIT-R scores comparable to Cluster 1. This pattern suggests that knowledge and risk perception alone may be insufficient to reduce problematic use. Awareness may fail to translate into behavior change when use is embedded in stress management or broader psychological vulnerability, and some consumers may partially implement or selectively disregard harm-reduction recommendations. Accordingly, consumers with high knowledge and risk awareness may still benefit from tailored support addressing coping, emotion regulation, and, when indicated, mental health care, rather than relying on education alone. An alternative interpretation is that Cluster 3 partly captures participants who use cannabis to manage subclinical somatic or psychological conditions (e.g., chronic pain, sleep difficulties, low-grade anxiety) that fell below the threshold of the study's exclusion criteria. This would be consistent with the profile's combination of frequent use, elevated risk awareness, and low endorsement of recreational MMM motives, which primarily capture hedonic and social drivers rather than therapeutic intent. Systematic assessment of somatic health indicators in future waves will be necessary to test this hypothesis.

Importantly, the distinction between Cluster 3 and Cluster 4 is not merely semantic. Cluster 3 corresponds to a low-motive but risk-aware profile, whereas Cluster 4 reflects a more globally disengaged profile, with minimal motives, lower safer-use knowledge, and very low perceived risk. This difference is further supported by their distinct external correlates: despite modest motive endorsement, Cluster 3 showed frequent use and problematic use levels comparable to Cluster 1, whereas Cluster 4 reported low use frequency and low problematic use, consistent with a casual, low-engagement pattern of cannabis use. Cluster 4, the largest group, reported minimal motives, low use frequency, and low problematic use (alongside Cluster 2), but also low safer-use knowledge and very low perceived risk. Members were older, initiated use later, and reported favorable income and health indicators, suggesting casual use with limited day-to-day relevance. While intensive intervention is unlikely to be needed, brief informational outreach may be warranted given low safer-use knowledge, particularly if use intensifies or shifts toward higher-potency products. The minimal endorsement of all cannabis use motives in Cluster 4 warrants further consideration. Rather than reflecting an absence of motivation, these low scores may suggest that the motives assessed by the MMM such as coping, enhancement, social, expansion, and conformity, do not fully capture the reasons why older, casual PWUC engage with cannabis. Alternative motivations such as habit, curiosity, trust in legally regulated products, sleep or physical comfort related motives, especially among older PWUC (which are not fully captured by the coping scale) may be more relevant for this subgroup, pointing to potential limitations of the MMM in capturing the full motivational spectrum of PWUC in regulated sales contexts.

It is worth noting that the cannabis-related harms discussed in the present study, such as problematic use, psychiatric symptoms, and reduced quality of life, are not necessarily causally attributable to cannabis use per se. The existing literature suggests that associations between cannabis use and adverse outcomes may be partially explained by third variables such as socioeconomic status, pre-existing mental health vulnerabilities, or co-occurring substance use (Leung et al., 2020, Taylor et al., 2019). The cross-sectional design of the present study precludes causal conclusions, and bidirectional associations between cannabis use and health outcomes cannot be ruled out. When interpreting perceived risk, it is also important to acknowledge that participants may have primarily considered direct pharmacological effects of cannabis rather than knowledge of statistical associations between use patterns and adverse outcomes, which may partly explain the modest relationship between risk perception and actual use patterns observed across profiles.

Overall, the profiles support a multidimensional view of risk under regulated access. Although between-cluster differences in outcomes are moderate, the profiles provide clinically actionable information. Comparable levels of problematic use and health outcomes may mask fundamentally different motivational and cognitive profiles, as illustrated by Clusters 1 and 3, which show similar CUDIT-R scores yet differ markedly in coping motives, risk awareness, and safer-use knowledge. These distinctions have direct implications for tailoring prevention and care. Perceived health risk showed only modest associations with use frequency and CUDIT-R scores, consistent with a partly independent subjective appraisal rather than a direct proxy for objective risk. This is compatible with evidence that risk perceptions and cannabis use may be linked through reciprocal or indirect pathways over time and may be shaped by social and legal-context factors.

More broadly, our findings align with evidence that cannabis-related harms concentrate in specific subgroups rather than being evenly distributed (Leung et al., 2020, Taylor et al., 2019). In our data, this high-harm minority is reflected in Cluster 1 and, to a lesser extent, Cluster 3, whereas Clusters 2 and 4 show comparatively low harms despite similar legal access. This pattern suggests that legalization does not homogenize risk and may amplify existing vulnerabilities among PWUC at risk for mental health conditions (Hall et al., 2023, Smart and Pacula, 2019). The persistence of heavy use and harms in Cluster 3 despite high knowledge and perceived risk also indicates that information-based interventions alone may be insufficient, particularly when use serves distress regulation. This aligns with evidence that educational programs can shift knowledge and perceived risk without consistently changing behavior (McBride, 2003, Harrison et al., 2024). In regulated markets, harm reduction may therefore need to combine structural measures (for example product standards, warning labels, pricing, and limits on high-potency products) with psychologically informed interventions targeted to higher-risk profiles. Future longitudinal analyses should examine whether profiles remain stable, how they evolve over time, and whether profile-tailored prevention and care improve outcomes.

## 8 Limitations and conclusion

The cross-sectional design precludes causal conclusions (Fischer et al., 2017), and the self-selected sample may not represent all PWUC (Cuttler et al., 2021, Hathaway et al., 2011). Self-report is subject to recall and desirability biases (Gmel et al., 2010). The use of single-item measures for perceived risk and health-related quality of life (EQ-5D VAS) represents a limitation, as these measures capture global appraisals rather than specific dimensions of risk or health. Future studies would benefit from including more multidimensional assessments of these constructs. Furthermore, although participants with severe medical conditions were excluded per eligibility criteria, subclinical somatic health conditions were not systematically assessed. Future research should examine the role of physical health status, including chronic pain and other somatic conditions, in shaping cannabis use motives and harm profiles within regulated sales frameworks, and assess how profiles change during follow-up as regulated access continues. In conclusion, the identified profiles provide a nuanced picture of PWUC diversity within regulated cannabis sales and support flexible, profile-sensitive approaches to prevention, harm reduction, and care.

## CRediT authorship contribution statement

**Rochat Lucien:** Writing – review & editing, Supervision, Methodology, Formal analysis. **Toso Laura:** Writing – review & editing, Data curation. **Aboulafia Brakha Tatiana:** Writing – review & editing. **Corino Tamara:** Writing – review & editing, Investigation, Data curation. **Dreifuss Bisson Maëlle:** Writing – review & editing, Writing – original draft, Visualization, Methodology, Investigation, Formal analysis, Data curation, Conceptualization. **Spierer Lucas:** Writing – review & editing, Supervision, Methodology, Funding acquisition. **Zullino Daniele:** Writing – review & editing, Funding acquisition. **Cattacin Sandro:** Writing – review & editing, Funding acquisition. **Seragnoli Federico:** Writing – review & editing.

## Fundings

This research is supported by the 10.13039/501100001711Swiss National Science Foundation (Grant #32513B_212616 to LS).

## Declaration of Competing Interest

The authors whose names are listed immediately below certify that they have NO affiliations with or involvement in any organization or entity with any financial interest (such as honoraria; educational grants; participation in speakers’ bureaus; membership, employment, consultancies, stock ownership, or other equity interest; and expert testimony or patent-licensing arrangements), or non-financial interest (such as personal or professional relationships, affiliations, knowledge or beliefs) in the subject matter or materials discussed in this manuscript.

## Figures and Tables

**Table:** 

**Variable**	**Median**	**IQR**	**Min**	**Max**
**Conformity**	5.0	0.0	5.0	25.0
**Product quality**	5.0	0.0	1.0	5.0
**Variety of choices**	4.0	2.0	1.0	5.0
**Ease of access**	4.0	2.0	1.0	5.0
**Curiosity about**	3.0	2.0	1.0	5.0
**the study**				
**Interest in the**	5.0	1.0	1.0	5.0
**results of the study**				
**Contribution to the development of new regulatory models**	5.0	1.0	1.0	5.0

**Table:** 

	**Cluster 1 (N = 128)**	**Cluster 2 (N = 345)**	**Cluster 3 (N = 379)**	**Cluster 4 (N = 438)**			
**Cluster profiling**	**M (SD)**	**M (SD)**	**M (SD)**	**M (SD)**	***F***	***p***	***η²part***
**Coping**	2.03 (1.06)^b,c,d^	-0.14 (0.64)^a,d^	-0.06 (0.80)^a,d^	-0.43 (0.57)^a,b,c^	394.94	< .001	.48 (large)
**Enhancement**	0.68 (0.87)^c,d^	0.76 (0.65)^c,d^	-0.16 (0.85)^a,b,d^	-0.66 (0.85)^a,b,c^	239.01	< .001	.36 (large)
**Social**	1.45 (1.13)^b,c,d^	0.57 (0.86)^a,c,d^	-0.30 (0.68)^a,b,d^	-0.61 (0.53)^a,b,c^	346.50	< .001	.45 (large)
**Expansion**	1.32 (0.85)^b,c,d^	0.53 (0.97)^a,c,d^	-0.38 (0.67)^a,b^	-0.48 (0.72)^a,b^	251.60	< .001	.37 (large)
**Knowledge about low-risk cannabis use**	-0.32 (1.11)^b,c^	0.02 (0.93)^a,c,d^	0.42 (0.81)^a,b,d^	-0.28 (1.04)^b,c^	42.56	< .001	.09 (moderate)
**Perception of risk**	-0.65 (0.56)^b,c,d^	0.00 (0.61)^a,c,d^	0.50 (0.65)^a,b,d^	0.20 (0.53)^a,b,c^	526.10	< .001	.51 (large)

*Note.* Numbers in the same row that do not share the same superscripts differ at p < .001. ^a^ Significantly different from Cluster 1. ^b^ Significantly different from Cluster 2. ^c^ Significantly different from Cluster 3. ^d^ Significantly different from Cluster 4. Effect sizes (η²) interpreted as small (.01), moderate (.06), or large (.14).

**Table:** 

	**1**	**2**	**3**	**4**	**5**	**6**
1. Coping	—					
2. Enhancement	.17	—				
3. Social	.33	.47	—			
4. Expansion	.35	.33	.39	—		
5. Knowledge (LRCU-K)	-.07	.08	-.05	-.09	—	
6. Perceived risk	.16	.01	.02	-.01	.21	—

*Note.* All values are Spearman's ρ. Knowledge =  knowledge of lower-risk cannabis use (LRCU-K); Perceived risk =  perceived health risk of current cannabis use. All correlations among the four motives (1–4) are positive and statistically significant (p < .001). Correlations between motives and knowledge or perceived risk are small and largely non-significant.

## Appendix C

***Spearman Correlations Among the Six Clustering Variables (N = 1290)***
